# Role of miR-1 and miR-133a in myocardial ischemic postconditioning

**DOI:** 10.1186/1423-0127-18-22

**Published:** 2011-03-16

**Authors:** Bin He, Jian Xiao, An-Jing Ren, Yu-Feng Zhang, Hao Zhang, Min Chen, Bing Xie, Xiao-Gang Gao, Ying-Wei Wang

**Affiliations:** 1 Department of Anesthesiology, Xinhua Hospital, Shanghai Jiaotong University School of Medicine, Kongjiang Road, Shanghai, China; 2 Department of Cardiothoracic Surgery, Changzheng Hospital, the Second Military Medical University, Fengyang Road, Shanghai, China; 3 Department of Pathophysiology, the Second Millitary Medical University, Xiangyin Road, Shanghai, China; 4 Department of Cardiothoracic Surgery, Changhai Hospital, the Second Millitary Medical University, Changhai Road, Shanghai, China; 5 Department of Cardiology, Shanghai Tenth People's Hospital, Tongji University, Middle Yanchang Road, Shanghai, China; 6 Department of Burn, Changhai Hospital, the Second Millitary Medical University, Changhai Road, Shanghai, China; 7 Department of Organ Transplantation, Changzheng Hospital, the Second Military Medical University, Fengyang Road, Shanghai, China

## Abstract

**Background:**

Ischemic postconditioning (IPost) has aroused much attention since 2003 when it was firstly reported. The role of microRNAs (miRNAs or miRs) in IPost has rarely been reported. The present study was undertaken to investigate whether miRNAs were involved in the protective effect of IPost against myocardial ischemia-reperfusion (IR) injury and the probable mechanisms involved.

**Methods:**

Thirty SD rats weighing 250-300 g were equally randomized to three groups: Control group, where the rats were treated with thoracotomy only; IR group, where the rats were treated with ischemia for 60 min and reperfusion for 180 min; and IPost group, where the rats were treated with 3 cycles of transient IR just before reperfusion. The extent of myocardial infarction, LDH and CK activities were measured immediately after treatment. Myocardial apoptosis was detected by TUNEL assay. The myocardial tissue was collected after IR or IPost stimulation to evaluate the miRNAs expression level by miRNA-microarray and quantitative real-time RT-PCR. Real-time PCR was conducted to identify changes in mRNA expression of apoptosis-related genes such as Bcl-2, Bax and Caspase-9 (CASP9), and Western blot was used to compare the protein expression level of CASP9 in the three groups. The miRNA mimics and anti-miRNA oligonucleotides (AMO) were transferred into the cultured neonatal cardiomyocytes and myocardium before they were treated with IR. The effect of miRNAs on apoptosis was determined by flow cytometry and TUNEL assay. CASP9, as one of the candidate target of miR-133a, was compared during IR after the miR-133a mimic or AMO-133a was transferred into the myocardium.

**Results:**

IPost reduced the IR-induced infarct size of the left ventricle, and decreased CK and LDH levels. TUNEL assay showed that myocardial apoptosis was attenuated by IPost compared with IR. MiRNA-microarray and RT-PCR showed that myocardial-specific miR-1 and miR-133a were down-regulated by IR, and up-regulated by IPost compared with IR. Furthermore, IPost up-regulated the mRNA expression of Bcl-2, down-regulated that of Bax and CASP9. Western blot showed that IPost also down-regulated the CASP9 protein expression compared with IR. The results of flow cytometry and TUNEL assay showed that up-regulation of miR-1 and miR-133a decreased apoptosis of cardiomyocytes. MiR-133a mimic down-regulated CASP9 protein expression and attenuated IR-induced apoptosis.

**Conclusion:**

MiRNAs are associated with the protective effect of IPost against myocardial IR injury. IPost can up-regulate miR-1 and miR-133a, and decrease apoptosis of cardiomyocyte. Myocardial-specific miR-1 and miR-133a may play an important role in IPost protection by regulating apoptosis-related genes. MiR-133a may attenuate apoptosis of myocardiocytes by targeting CASP9.

## Background

Both percutaneous coronary intervention (PCI) and coronary artery bypass graft (CABG) are effective for myocardial infarction (MI) [1]. However, ischemia reperfusion (IR) induced by revascularization may contribute to subsequent myocardial injury, in which apoptosis may play a key role in myocardial IR injury [2]. It is therefore important to find the endogenous protective mechanism against apoptosis induced by myocardial IR injury.

It has been proved that both ischemia preconditioning (IPre) and ischemic postconditioning (IPost) have protective effects against subsequent prolonged myocardial IR injury [3-5]. With an unpredicted onset of myocardial ischemia, IPre is inconvenient to perform for clinical protection treatment. Unlike IPre, IPost is induced after ischemia, and can be easily performed in cardiac operations. Therefore, IPost has aroused much attention [4-6] since 2003 when it was firstly reported by Zhao *et al. *IPost has been reported to reduce infarct size, prevent heart failure, and attenuate tumor necrosis factor-α (TNF-α) [7-9]. Recently, more studies have reported that IPost could reduce apoptosis of cardiomyocytes not only in animal experiments but also in patients undergoing PCI [10-13].

Recently, microRNAs (miRNAs or miRs) have been demonstrated to play an important role in myocardial injury. For example, miR-208 was up-regulated, while miR-1 and miR-133a were down-regulated in MI [14]. MiR-1 and miR-133 produced opposing effects on apoptosis induced by H_2_O_2 _[15]. MiR-320 was down-regulated, while miR-21, miR-146b and miR-491 were up-regulated after IR injury [16]. MiR-199a was down-regulated by hypoxia preconditioning in cardiomyocytes [17]. Among the miRNAs, miR-1 and miR-133 are specifically expressed in cardiac and skeletal muscles [14,15].

However, the role of miRNAs in IPost has rarely been reported. The present study was undertaken to see whether miRNAs, especially myocardial-specific miR-1 and miR-133a, were involved in the protective effect of myocardial IPost by regulating apoptosis-related genes.

## Materials and methods

### Animal care

All animal experiments were approved by the Animal Research Ethics Committee of the Second Military Medical University, Shanghai, China.

### In vivo rat model

SD rats (250-300 g) were anesthetized with 10% chloral hydrate (300 mg/kg, i.p.) before endotracheal intubation. IR was induced by ligating the left anterior descending artery (LAD) for 60 min, followed by loosening the ligature for 180 min [18]. Successful ligation of LAD was evidenced by immediate regional cyanosis in the anterior ventricular wall and the apex of the heart with color change greater than 40% of the left ventricle (LV) and confirmed by electrocardiography (ECG).

### Experimental protocols

Thirty rats were equally randomized to three groups: Control group (Con group, n = 10), where the rats underwent thoracotomy without ligation; IR group (n = 10), where the rats were treated with ischemia for 60 min and reperfusion for 180 min; and IPost group (n = 10), where 3 cycles of transient IR (ischemia 30 sec/reperfusion 30 sec) were given just before reperfusion. This sample size was chosen based upon the results of a power analysis.

### Infarct size measurement

Infarct size of the myocardium was measured as previously described [19]. Total left ventricular area (LV), infarct area (INF) and area at risk (AAR) were determined by computerized planimetry. The percentage of the INF/LV, AAR/LV and INF/AAR was calculated.

### LDH and CK assay

Blood serum was collected after 180 min reperfusion for determination of lactate dehydrogenase (LDH) and creatine kinase (CK) activities.

### TUNEL assay in vivo

Terminal dUTP nick end-labeling (TUNEL) assay was performed as previously described [20]. Nuclei were counted in 10 microscopic fields from the midventricular section (from the apex to the ligation level) of each heart. The average of the TUNEL-positive nuclei ratio in 10 microscopic fields was calculated to compare the apoptosis ratio within the different groups.

### MiRNA-microarray and quantitative real-time RT-PCR of miRNA and apoptosis-related genes

Total RNA of cells was isolated by using TRIzol reagent, and reverse transcribed according to the manufacturer's instructions (Fermentas, in CA).

MiRNA expression profiling was determined by miRNA-microarray analysis (LC Sciences Inc). Dysregulated miR-1 and miR-133a were validated by quantitative real-time RT-PCR in duplicates using Rotor Gene 3000 (Corbett Research, Sydney, Australia). The annealing temperature of miRNA-1 and miRNA-133a was set at 60°C, and that of Bcl-2 and Bax was set at 58°C. The comparative Ct (threshold cycle) method with arithmetic formulae (2^-ΔΔCt^) was used to determine relative quantitation of gene expression of both target and housekeeping genes (βactin). The primers of miRNAs and apoptosis-related genes (Bcl-2, Bax and CASP9) used in the study are shown in Table 1.

**Table 1:** Primers used for quantitative real-time RT-PCR

RT-primers	miR-1	5'-GGCTGCCGACCGTGTCGTGGAGTCGGCAATTGGTCGGCAGCCATACACAC-3'
	miR-133a	5'-GTCGTATCCAGTGCAGGGTCCGAGGTATTCGCACTGGATACGACACAGCT-3'
PCR-primer	miR1-F	5'-CTGTCACTCGAGCTGCTGGAATG-3'
	miR1-R	5'-ACCGTGTCGTGGAGTCGGCAATT-3'
	miR133a-F	5'-CTGCATTGGTCCCCTTCAAC-3'
	miR133a-R	5'-CAGTGCAGGGTCCGAGGTAT-3'
	β actin-R	5'-ATGGTGGGTATGGGTCAGAAGG-3'
	β actin-F	5'-TGGCTGGGGTGTTGAAGGTC-3'
	Bcl2-F	5'-CGGGAGAACAGGGTATGA-3'
	Bcl2-R	5'-CAGGCTGGAAGGAGAAGAT-3'
	Bax-F	5'-GTTGCCCTCTTCTACTTTGC-3'
	Bax-R	5'-ATGGTCACTGTCTGCCATG-3'
	CASP9-F	5'-ATTGGCGACCCTGAGAAG-3'
	CASP9-R	5'-CCAGATGCTGTCCCATACC-3'

### Western blot analysis in vivo

The protein expression of CASP9 was detected by Western blot analysis as previously described [21].

### mimics and anti-miRNA oligonucleotides (AMOs) of miRNAs synthesis

miRNA's mimics (Gene Bank NO.: rno-miR-1, NR 032116.1; rno-mir-133a, NR 031879.1) and AMOs (AMO-1 and AMO-133a) were synthesized by Jima Inc (Shanghai, China). The sequences of miRNA mimics and AMOs are showed in Table 2.

**Table 2:** The sequences of miRNA mimics and AMOs

miR-1 mimic	5'-UGGAAUGUAAAGAAGUGUUAUACACACUUCUUUACAUUCCAUU-3'
AMO-1	5'-AUACACACUUCUUUACAUUCCA-3'
miR-133a mimic	5'-UUUGGUCCCCUUCAACCAGCUGGCUGGUUGAAGGGGACCAAAUU-3'
AMO-133a	5'-CAGCUGGUUGAAGGGGACCAAA-3'

### Mimic and AMO of miRNA pretreatment in vivo

Mimic and AMO of miRNA pretreatment in vivo were performed as previously described [22]. With the chest open as described above, 100 ul synthesized miR-133a mimic or AMO-133a (50 mg/Kg), pretreated with lipofectamine 2000 (Invitrogen), was injected into the myocardium. Ten sites were selected on the LV anterior wall for intramuscular injection. The chest was closed after injection and the rat was allowed to recover. IR treatment was performed 48 h later.

### Cell culture, Mimic and AMO of miRNA pretreatment in vitro

Neonatal cardiomyocytes were prepared from the heart of SD rats younger than 3 days. The isolated cardiomyocytes were obtained and cultured by the method reported by Sadoshima *et al *[23]. On the 3rd day, the cardiomyocytes were treated with 24 h hypoxia (3%O_2_, 5%CO_2_, 92%N_2_) and 3 h reoxygenation (5%CO_2_, 95%air). To demonstrate the effect of miR-1 and miR-133a on IR-induced apoptosis of cardiomyocytes, miRNA's mimics and AMOs (50 nM) were transferred into the cardiomyocytes with lipofectamine 2000 (Invitrogen) 48 h before IR.

### Flow cytometry analysis of apoptosis by annexin V/PI staining

Neonatal cardiomyocytes were stained by annexin V/PI as previously described [24], and finally analyzed with a flow cytometer (Becton-Dickinson, USA) at excitation 488 nm and emission 615 nm according to the manufacturer's instructions.

### Statistical analysis

Quantitative data are presented as mean ± standard error. Statistical significance was determined using one-way ANOVA. *P *< 0.05 was considered statistically significant.

## Results

### IPost produces cardioprotective effects against IR injury

**Figure 1:**
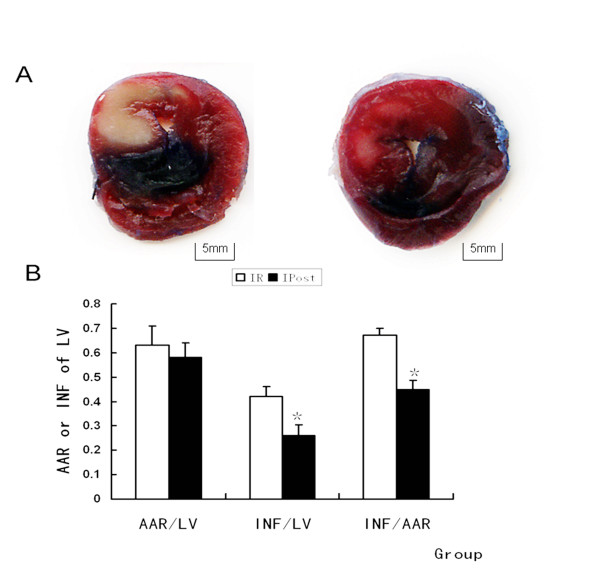
**IPost reduced the IR-induced infarct size of LV**. (A) Representative mid-myocardial crosssections of TTC-stained hearts for IR and IPost. Dark blue area, nonischemic zone; remaining area, AAR; white area, infracted tissue; red area, viable myocardium. (B) AAR/LV was similar between IR and IPost groups. IPost significantly attenuated myocardial INF/LV and INF/AAR compared with IR (n = 10, *P < 0.05, compared with IR group).

**Figure 2:**
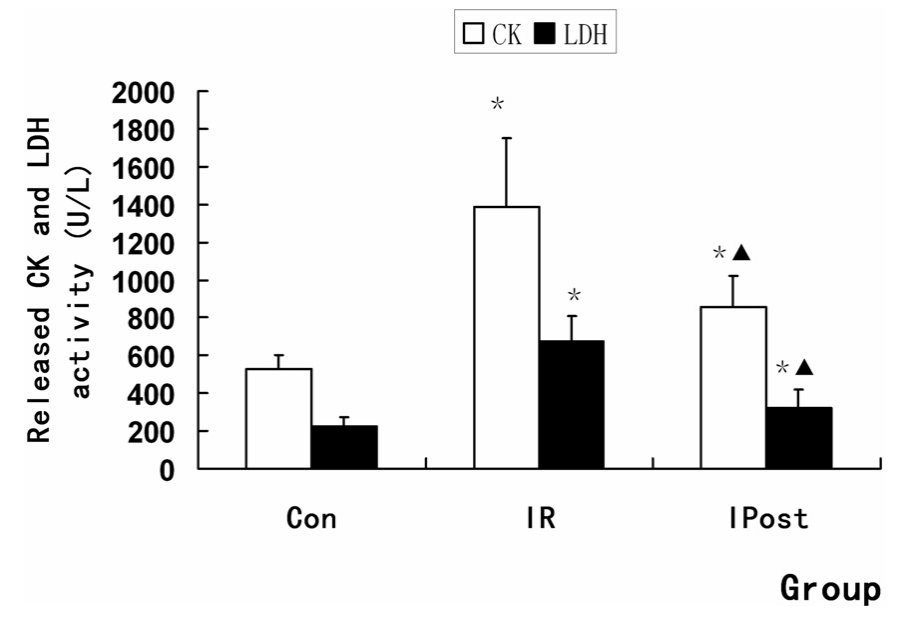
**LDH and CK assay of blood serum**. The activities of CK and LDH were increased by IR, and IPost decreased them compared with IR (n = 10, *P < 0.05, compared with Con group; ^▲^P < 0.05, compared wit IR group).

The extent of myocardial infarction was evaluated after reperfusion. Representative photographs of midventricular cross sections of evans blue and TTC-stained hearts were taken from Control, IR and IPost groups. AAR/LV was similar between IR and IPost groups (*P *> 0.05), while IPost significantly attenuated myocardial INF/LV and INF/AAR compared with IR (*P < 0.05*, Figure 1). IPost also decreased circulating CK and LDH levels significantly (*P *< 0.05, Figure 2).

### IPost attenuates the myocardiocytes apoptosis induced by IR

**Figure 3:**
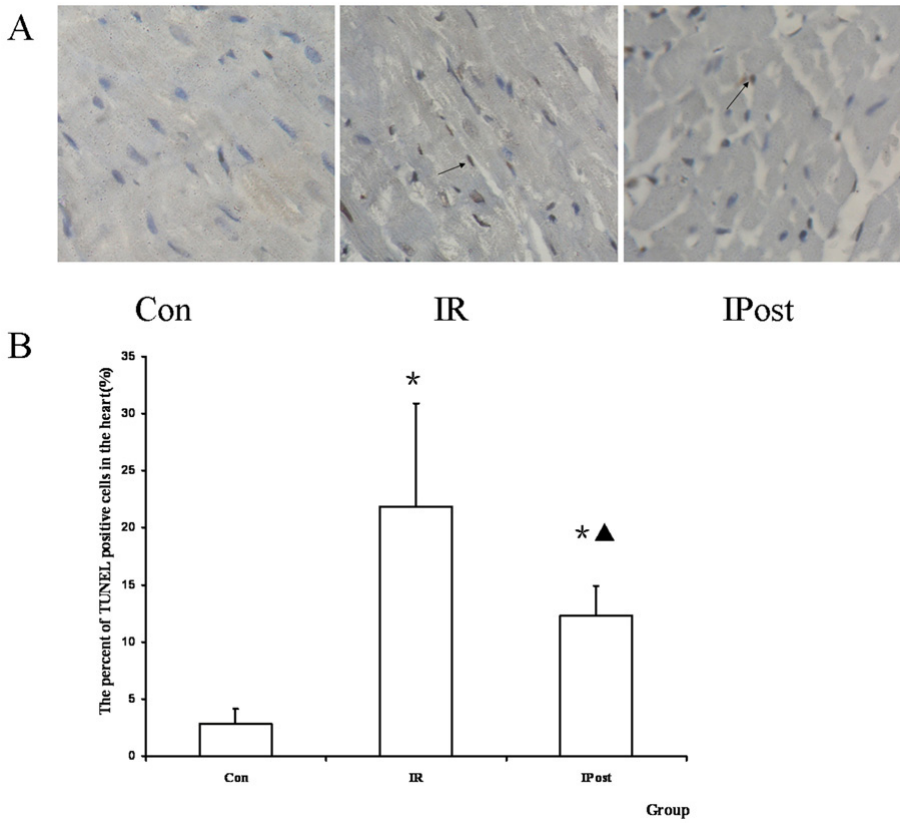
**TUNEL assay**. (A) TUNEL staining pictures, in which brown staininged cells were TUNEL positive cells (magnification, × 400). (B) The percent of TUNEL positive cells in the heart. TUNEL positive cells were increased by IR, and decreased by IPost (n = 10, *P < 0.05, compared with Con group; ^▲^P < 0.05, compared wit IR group).

TUNEL assay was performed to quantitate the apoptosis *in vivo*. It was found that TUNEL staining positive cells were increased by IR, and were decreased by IPost (*P *< 0.05, Figure 3).

### MiRNAs are dysregulated in the rat myocardium by IR injury

**Figure 4:**
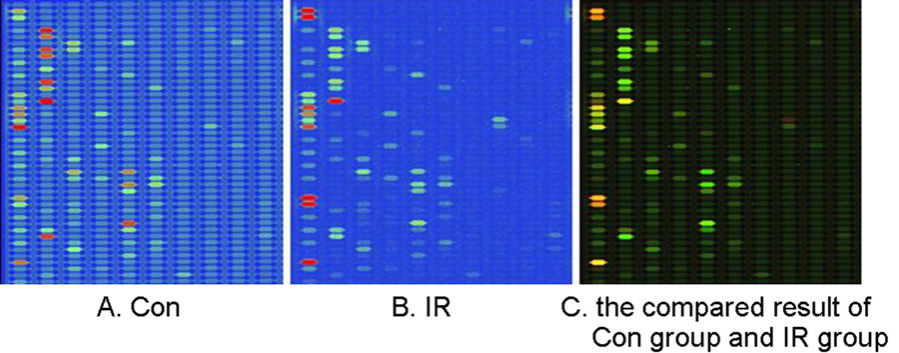
**MiRNA-microarray compaired between Control and IR groups**. 16 miRNAs were dysregulated by IR, of which 10 miRNAs were up-regulated and the other 6 miRNAs were down-regulated significantly. The green signal is labeled with cy5 and the red signal was labeled by cy3 (green: cy3 >cy5; yellow: cy3 = cy5; red: cy3 <cy5).

To compare the expression of miRNAs between Control and IR groups, miRNA-microarray analysis was used to determine miRNAs level in the rat heart. It was found that many miRNAs were significantly dysregulated by IR injury (Figure 4, Table 3).

**Table 3:** MiRNAs significantly dysregulated by IR

**No**.	Probe_ID	Control group Signal	IR group Signal	IR group/Control group
1	rno-miR-21	109.74	701.76	6.39
2	rno-miR-26b	381.85	1,850.99	4.85
3	rno-miR-499	40.42	195.93	4.85
4	rno-miR-214	2,104.89	500.29	0.24
5	rno-miR-125b-5p	2,826.12	1,000.25	0.35
6	rno-miR-126	3,863.56	9,309.76	2.41
7	rno-miR-1	51,964.34	24,454.18	0.47
8	rno-let-7e	757.45	1,479.82	1.95
9	rno-miR-23a	2,395.29	4,881.28	2.04
10	rno-miR-133a	4,705.42	2,362.14	0.50
11	rno-miR-133b	4,009.39	2,077.02	0.52
12	rno-miR-24	2,190.29	1,234.00	0.56
13	rno-miR-23b	3,076.83	5,356.80	1.74
14	rno-let-7d	5,441.08	7,003.94	1.29
15	rno-miR-26a	6,997.25	8,860.27	1.27
16	rno-let-7a	8,879.37	11,121.66	1.25

### IPost regulates miRNA expression

**Figure 5:**
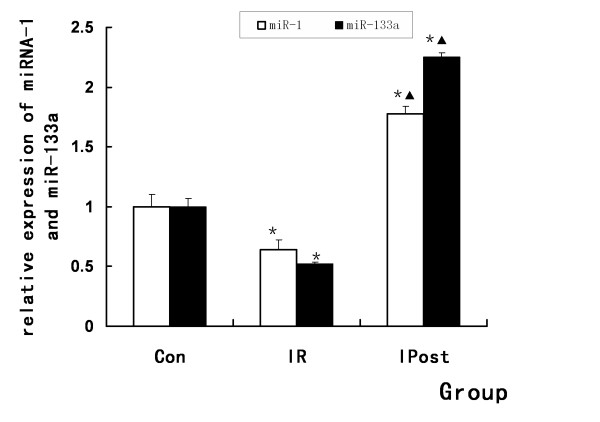
**Regulation of miR-1 and miR-133a by IPost**. MiR-1 and miR-133a were down-regulated in IR group, while IPost up-regulated them as compared with IR group (n = 10, *P < 0.05, compared with Con group; ^▲^P < 0.05, compared wit IR group).

To further validate the results of microarray analysis and confirm the effect of IPost on miRNAs, quantitative real-time RT-PCR was used to detect miRNAs expression levels in Control, IR and IPost groups. It was found that myocardial-specific miR-1 and miR-133a were down-regulated after IR. IPost up-regulated miR-1 and miR-133a compared with IR (*P *< 0.05, Figure 5).

### IPost regulates apoptosis-related genes

**Figure 6:**
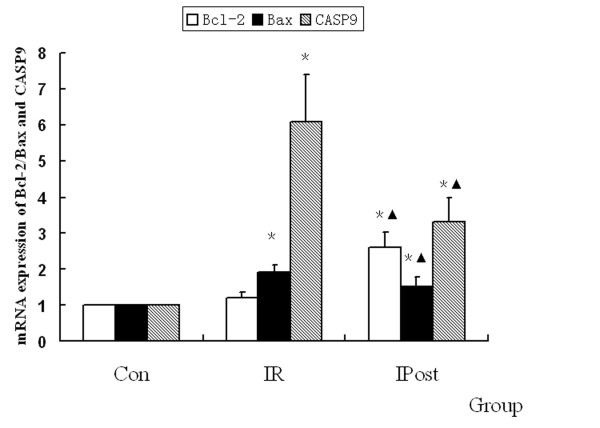
**Regulation of apoptosis-related gene mRNA by IPost**. Compared with Control group, IR increased the mRNA expression of Bax and CASP9. While IPost increased Bcl-2 mRNA expression, and decreased Bax mRNA expression (n = 10, *P < 0.05, compared with Con group; ^▲^P < 0.05, compared with IR group).

To demonstrate the effect of IPost on IR-induced apoptosis, quantitative real-time PCR was used to detect the mRNA expression of Bcl-2, Bax and CASP9, which were regarded as the marker of apoptosis. It was found that Bcl-2, Bax and CASP9 were up-regulated by IR, but there was no significant difference in Bcl-2 expression compared with Control group (*P *> 0.05). IPost decreased the mRNA expression of Bax and CASP9, and increased Bcl-2 mRNA level as compared with IR group (*P *< 0.05, Figure 6).

### IPost regulates the protein expression of CASP9

**Figure 7:**
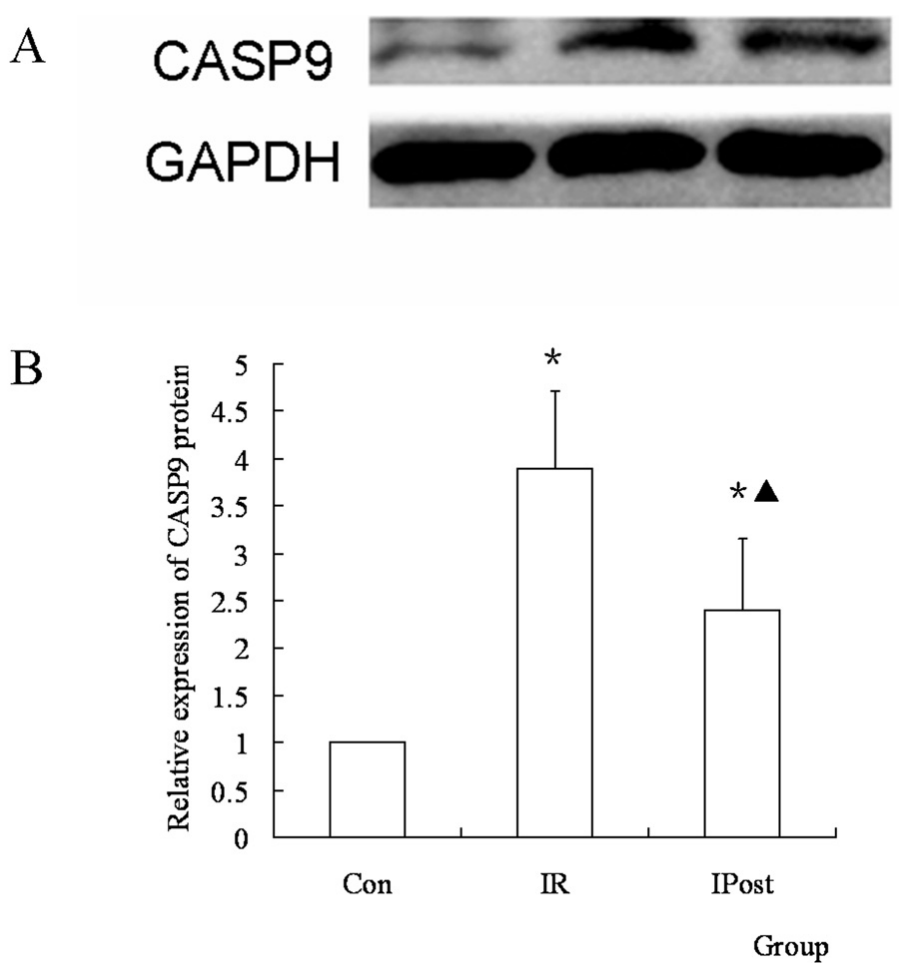
**The protein expression of CASP9 was regulated by IPost**. (A) Western blot of CASP9 in different groups. (B) The relative quantity of CASP9 protein in different groups. IR up-regulated CASP9 protein compared with Con group, and IPost down-regulated CASP9 protein compared with IR. (n = 10, *P < 0.05, compared with Con group; ^▲^P < 0.05, compared with IR group)

To determine the effect of IPost on CASP9 protein during IR, the protein expression of CASP9 in different groups was determined by Western blot. It was found that the protein expression of CASP9 was up-regulated in IR group compared with Control group, and it was down-regulated in IPost group compared with IR (*P *< 0.05, Figure 7).

### MiR-133a regulates the protein expression of CASP9

**Figure 8:**
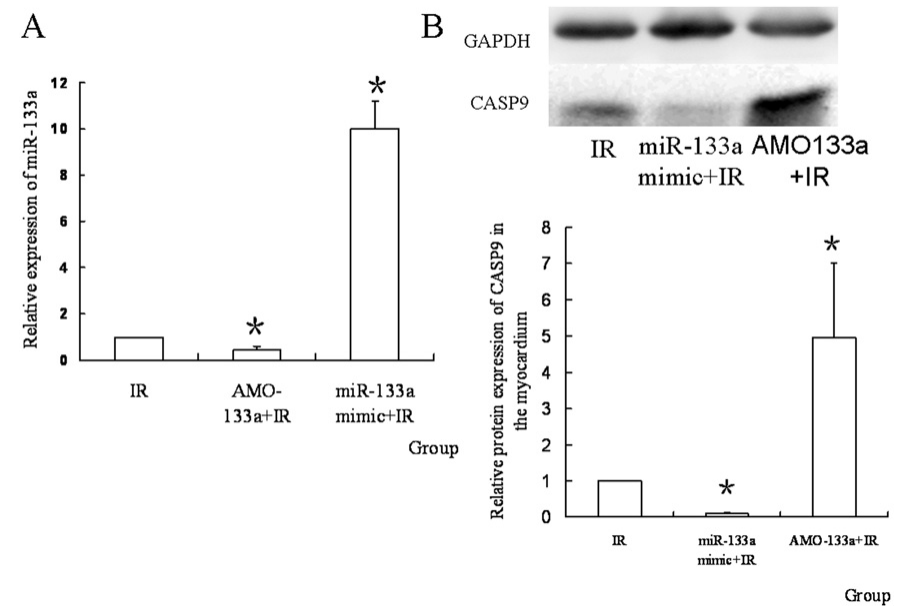
**The expression of miR-133a and CASP9 protein after transferring the mimic or AMO**. (A) Relative expression of miR-133a in different groups. MiR-133a was down-regulated by AMO-133a, and up-regulated by miR-133a mimic (n = 10, *P < 0.05, compared with IR group; ^▲^P < 0.05, compared with AMO-133+IR group); (B) The relative quantity of CASP9 protein in different groups. AMO-133a up-regulated CASP9 protein, and miR-133a mimic down-regulated it(n = 10, *P < 0.05, compared with IR group).

To see whether miR-133a regulated the CASP9 protein during IR, miR-133a mimic or AMO-133a was transferred into the myocardium before IR. It was found that the expression of CASP9 protein was uperegulated by AMO-133a and down-regulated by miR-133a mimic (*P *< 0.05, Figure 8).

### MiR-133a mimic attenuates apoptosis of myocardiocytes in vivo

**Figure 9:**
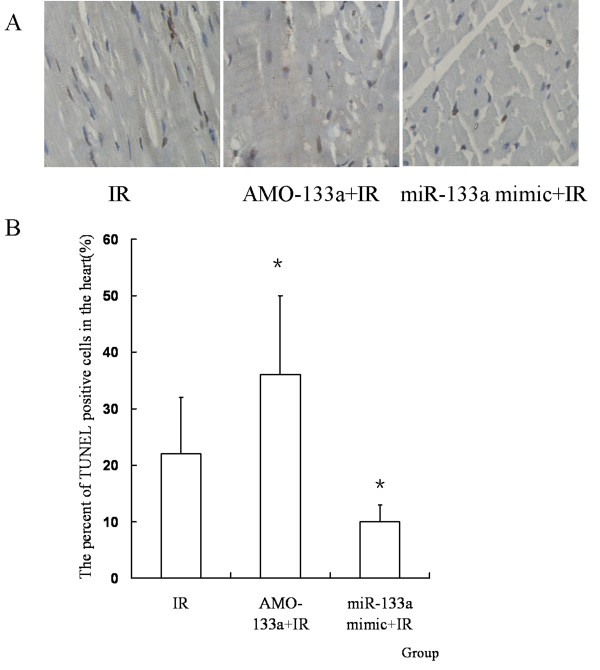
**MiR-133a mimic attenuates myocardiocyte apoptosis in vivo**. (A) TUNEL staining pictures, in which brown stained cells were TUNEL positive cells (magnification, × 400). (B) The percent of TUNEL positive cells in the heart. MiR-133a mimic decreased the apoptosis ratio induced by IR, while AMO-133a increased the apoptosis ratio (n = 10, *P < 0.05, compared with IR group).

To see whether miR-133a regulated cell apoposis induced by IR *in vivo*, miR-133a mimic or AMO-133a was transferred into the myocardium before IR. It was found that miR-133a mimic decreased the apoptosis ratio induced by IR, while AMO-133a increased the apoptosis ratio (*P *< 0.05, Figure 9).

### MiRNA-1 and miRNA-133a regulate apoptosis of cardiomyocytes

**Figure 10:**
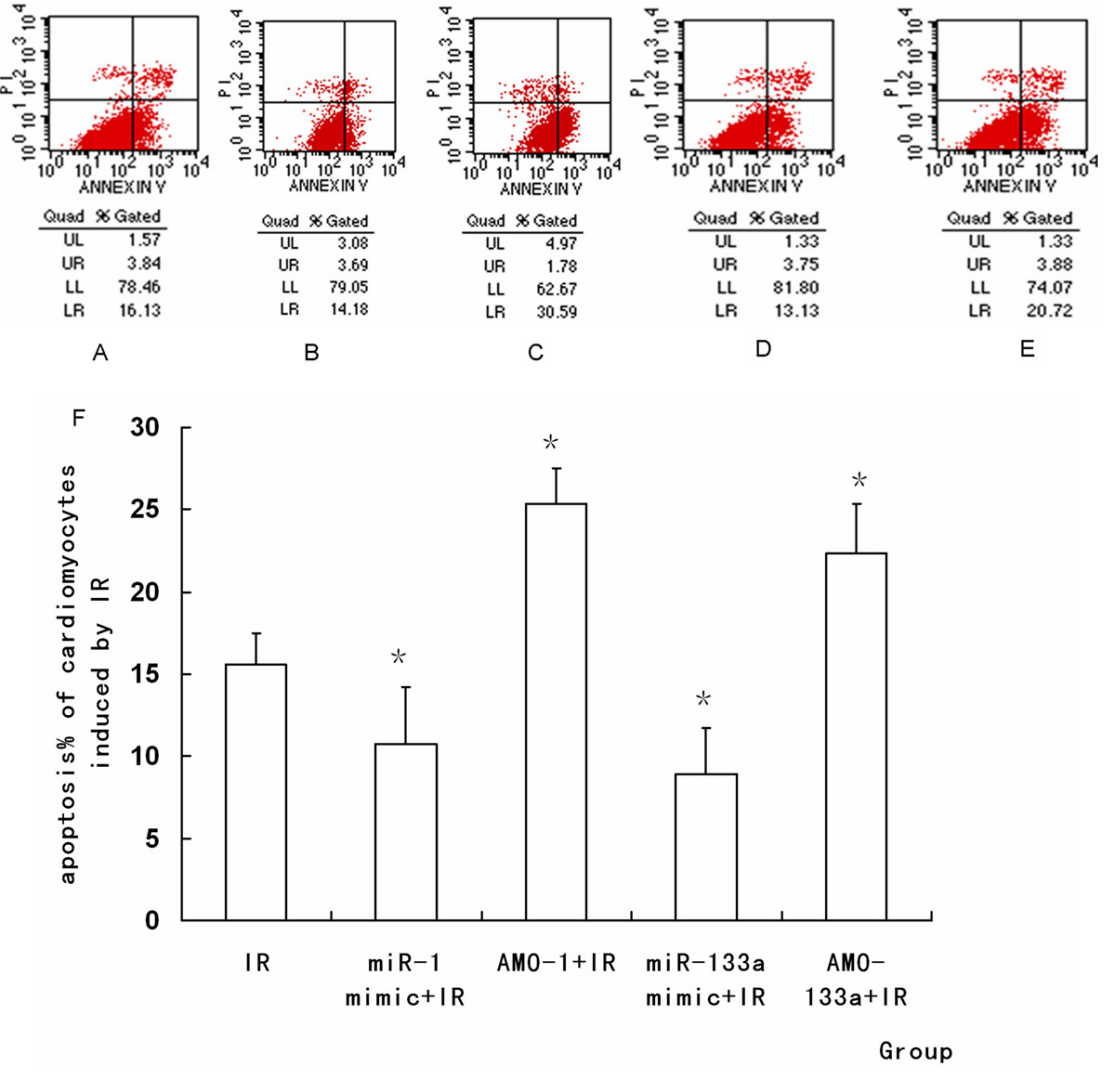
**Representative diagrams of the flow cytometric readings for myocardiocytes stained with annexin V and propidium iodide (PI)**. **(**A) IR. (B) miR-1 mimic+ IR. (C) AMO-1 +IR. (D) miR-133a mimic+ IR. (E) AMO-133a inhibitor +IR. (F) The percentage of apoptosis induced by IR in each group. MiR-1 promoted cell aopoptosis during IR, but miR-133a inhibited cell apoptosis during IR. (*P < 0.05, compared with IR group compared with IR group)

The apoptotic percentage (AP) was determined by flow cytometry. Treatment with miR-1 or miR-133a mimic significantly decreased AP of cardiomyocytes induced by IR, while IR-induced apoptosis was increased by AMO-1 or AMO-133a pretreatment. These results indicated that miR-1 and miR-133a had a cytoprotective effect against IR-induced apoptosis (*P *< 0.05, Figure 10).

## Discussion

Cardiomyocyte apoptosis is a key event in IR hearts. IPost has been demonstrated to have a protective effect against IR-induced apoptosis. We also found that IPost reduced INF of LV, and decreased LDH and CK activities. Many genes are known to be dysregulated by IR [25]. Studies have demonstrated that Bcl-2, Bax and CASP9 are apoptosis-related genes. Bcl-2 can attenuate apoptosis, while Bax can promote apoptosis [2,26,27]. We found that IPost attenuated the mRNA expression of Bax and CASP9, and increased Bcl-2 mRNA level as compared with IR. We also found that the protein expression of CASP9 was down-regulated in IPost group compared with IR. It was found in our study that TUNEL staining positive cells were increased by IR, and decreased by IPost. We presumed that IPost might attenuate apoptosis induced by IR. But how the expression of apoptosis-related genes was regulated remains uncertain.

MiRNAs are endogenous regulators of gene expression, and have been demonstrated to be involved in cardiac IR injury. Some miRNAs could reduce myocardial infarction through repressing apoptotic genes and up-regulating anti-apoptotic genes [15]. Many apoptosis-related genes, such as ET1, Caspases and HSPs, were target genes of the miRNAs. According to the bioinformatics of Targetscan, CASP9 was a potential target of miR-133a. This study manifested that miRNAs could serve as molecular switches to trigger an immediate change in apoptosis-related gene expression in response to IPost. To the best of our knowledge, the present study for the first time demonstrated the miRNA expression signature in IPost hearts.

By using miRNA-microarray analysis, the present study compared IR-injured rat hearts and normal rat hearts, and found that 16 miRNAs were dysregulated by IR, of which 10 microRNAs were up-regulated and the other 6 microRNAs were down-regulated. Among these miRNAs, miR-1 was down-regulated by IR, which is consistent with other reports [14,28]. We also found that miR-1 was up-regulated by IPost compared with IR, which is consistent with other reports of miR-1 regulated by IPre or heat-shock pretreatment [22,29,30]. MiR-1 is a myocardial-specific miRNA, which has been demonstrated to be associated with apoptosis-related genes such as heat shock protein (HSP), and indirectly regulate eNOs. It was reported that IPre up-regulated miR-1, miR-21 and miR-24, and the protein expression of HSP70 was up-regulated by pretreatment of these miRNAs. Furthermore, not only IPre but also heat-shock pretreatment, which can protect the heart against IR injury, could up-regulate miR-1 [22,30]. But conflicting results were reported in other studies. It was reported that the level of miR-1 was increased in response to oxidative stress [15].

It was found in our study that IPost up-regulated miR-1 and attenuated IR-induced INF together with dysregulating apoptosis-related gene, suggesting that IPost may protect the myocardium during IR by up-regulating miR-1, and then regulated apoptotic genes indirectly. We transferred the mimic and AMO of miR-1 into the cardiomyocytes 48 h before IR, and found that miR-1 mimic attenuated cell apoptosis, and AMO-1 increased apoptosis, as shown by flow cytometry. So we think that miR-1 may protect cardiomyocytes against IR through regulating some apoptosis-related genes.

We also found that miR-133a was down-regulated by IR and up-regulated by IPost, which is consistent with other reports [14,15,28,31]. MiR-133a and miR-1 are clustered on the same chromosome loci and transcribed together in a tissue-specific manner [32]. MiR-133a is essential in orchestrating cardiac development [33]. MiR-133a can also regulate cardiac rhythms by targeting HCN2 and HCN4 [34]. It was reported that miR-133 exhibited an anti-apoptotic effect in IR by regulating the expression of CASP9 [15]. CASP9 was not only the potential target protein of miR-133a but the important pro-apoptotic factor during IR [35]. So we selected CASP9 as the potential target protein of miR-133a to see whether miRNA was involved in the anti-apoptotic effect of IPost against IR injury. It was found that IPost enhanced the expression of miR-133a during IR, and that CASP9 protein was up-regulated by IR and down-regulated by IPost. In addition, CASP9 protein was down-regulated by miR-133a mimic and up-regulated by AMO-133a. After transferring miR-133a mimic and AMO-133a into the cultured neonatal cardiomyocytes and myocardium, we found that miR-133a mimic attenuated apoptosis, and AMO-133a promoted apoptosis, as shown by flow cytometry and TUNEL. We therefore speculate that miR-133a has a protective effect against IR, and can attenuate myocardiocyte apoptosis by targeting CASP9, and that IPost can enhance miR-133a expression to reduce cardiomyocyte apoptosis.

## Conclusion

In summary, our results confirm that myocardial-specific miR-1 and miR-133a play an important role in IPost protection against myocardial IR injury by regulating apoptosis-related genes. The most significant findings are up-regulation of miR-1 and miR-133a in IPost compared with IR hearts. And up-regulation of miR-1 and miR-133a can decrease cardiomyocyte apoptosis. We found that CASP9 was a potential target of miR-133a. IPost down-regulated CASP9 compared with IR, while miR-133a mimic down-regulated CASP9 protein and attenuated cardiomyocyte apoptosis induced by IR. The goal of our ongoing research is to seek other target genes of miRNAs involved in the mechanisms of myocardial Ipost protection.

## Competing interests

The authors declare that they have no competing interests.

## Authors' contributions

BH and JX performed the major experiments and analyzed the data. AJR participated in the design of the study and data interpretation. YFZ, HZ, MC and XGG participated in part of the experiments. BX participated in the data interpretation and manuscript improvement. YWW designed the experiments, interpreted the data and wrote the manuscript. All authors read and approved the final manuscript.
